# A New Look at the Functional Organization of the Golgi Ribbon

**DOI:** 10.3389/fcell.2019.00171

**Published:** 2019-08-21

**Authors:** Jaakko Saraste, Kristian Prydz

**Affiliations:** ^1^Department of Biomedicine and Molecular Imaging Center, University of Bergen, Bergen, Norway; ^2^Department of Biosciences, University of Oslo, Oslo, Norway

**Keywords:** Golgi ribbon, mitosis, cell migration, cell differentiation, Golgi bypass, centrosome, intermediate compartment, recycling endosome

## Abstract

A characteristic feature of vertebrate cells is a Golgi ribbon consisting of multiple cisternal stacks connected into a single-copy organelle next to the centrosome. Despite numerous studies, the mechanisms that link the stacks together and the functional significance of ribbon formation remain poorly understood. Nevertheless, these questions are of considerable interest, since there is increasing evidence that Golgi fragmentation – the unlinking of the stacks in the ribbon – is intimately connected not only to normal physiological processes, such as cell division and migration, but also to pathological states, including neurodegeneration and cancer. Challenging a commonly held view that ribbon architecture involves the formation of homotypic tubular bridges between the Golgi stacks, we present an alternative model, based on direct interaction between the biosynthetic (pre-Golgi) and endocytic (post-Golgi) membrane networks and their connection with the centrosome. We propose that the central domains of these permanent pre- and post-Golgi networks function together in the biogenesis and maintenance of the more transient Golgi stacks, and thereby establish “linker compartments” that dynamically join the stacks together. This model provides insight into the reversible fragmentation of the Golgi ribbon that takes place in dividing and migrating cells and its regulation along a cell surface – Golgi – centrosome axis. Moreover, it helps to understand transport pathways that either traverse or bypass the Golgi stacks and the positioning of the Golgi apparatus in differentiated neuronal, epithelial, and muscle cells.

## Introduction

**FIGURE 1:**
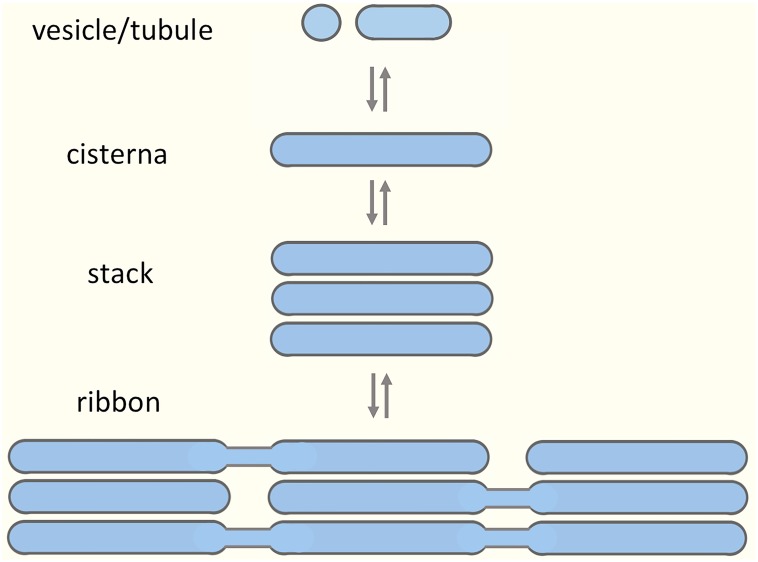
Building blocks of the Golgi apparatus. A model suggesting modular assembly and disassembly of the Golgi apparatus, based on its organization in various cell types and during different stages of the cell cycle. The prevailing view is that the preformed Golgi stacks in mammalian cells extend tubules that undergo tethering and fusion, thereby giving rise to a continuous Golgi ribbon consisting of compact (stacked) and non-compact (tubular) regions. Here, we argue that the non-compact zones are structurally more complex, being occupied by pleiomorphic “linker compartments”, which due to their function in the biogenesis of the Golgi stacks also dynamically join them together.

The Golgi apparatus modifies, sorts and transports proteins, lipids, and complex carbohydrates at the crossroads of the secretory and endocytic pathways. The Golgi is structurally unique, consisting of polarized stacks of flattened *cisternae* flanked by tubular networks (Mellman and Simons, 1992; Weidman et al., 1993; Mollenhauer and Morré, 1998; Jackson, 2009). Two opposing hypotheses have been put forward to explain the formation of such complex architecture (Glick, 2002). According to a more traditional view, the biogenesis of the Golgi stacks requires a permanent template; however, the nature of such a template has not been unequivocally established (Palade, 1983; Seemann et al., 2000). According to another proposition, the Golgi apparatus is a self-organizing structure, which assembles from dynamic components, exists in a state of equilibrium, and is capable of *de novo* formation (Misteli, 2001; Altan-Bonnet et al., 2004; Ronchi et al., 2014). In addition, there is data suggesting that the Golgi apparatus is a modular structure, with the joining of cisternal stacks into a ribbon structure representing the highest order of assembly (Nakamura et al., 2012; Figure 1). Evidence for structural Golgi modules may be obtained when looking more closely at different cell types or dividing cells. For example, during mitosis the Golgi stacks undergo disassembly, and resident Golgi enzymes temporarily end up in a vesicular Golgi haze (Shorter and Warren, 2002; Marie et al., 2012). The budding yeast *Saccharomyces cerevisiae* is generally considered to contain separate Golgi *cisternae* (Suda and Nakano, 2012); however, formation of stacked Golgi-like structures is observed in mutant yeast cells or under certain growth conditions (Rambourg et al., 1993; Hashimoto et al., 2002). Most typically, invertebrates, plants and many fungi contain individual or pairs of Golgi stacks distributed throughout the cytoplasm close to ER exit sites (ERES). Vertebrate cells display the highest level of complexity as they contain a Golgi ribbon, consisting of numerous cisternal stacks (compact zones) connected by tubular networks (non-compact zones) into a single copy organelle (Ladinsky et al., 1999; Kepes et al., 2005).

However, why vertebrate cells build a Golgi ribbon has generally remained an enigma (Wei and Seemann, 2010; Gosavi and Gleeson, 2017). Namely, ribbon organization is not strictly required for secretion, as clearly demonstrated by experiments with nocodazole, a microtubule (MT)-depolymerizing drug, which causes the replacement of the central Golgi ribbon by ERES-associated ministacks (Cole et al., 1996a; Thyberg and Moskalewski, 1999; Fourriere et al., 2016). It has been suggested that ribbon organization, by allowing lateral mobility of Golgi enzymes between the stacks, ensures correct glycosylation of cargo proteins (Puthenveedu et al., 2006; Xiang et al., 2013). Based on a rim progression Golgi model, lateral connections between neighboring stacks may facilitate anterograde intra-Golgi transport of large-sized cargo proteins (Lavieu et al., 2014), or allow the formation of large aggregates of endothelial von Willebrand factor (Ferraro et al., 2014). This proposal is in accordance with super-resolution light microscopy (LM) of individual Golgi stacks, showing the preferential localization of bulky, but not small cargo proteins to cisternal rims. Moreover, a large number of cargo processing enzymes localize to the central portion of the *cisternae*, while transport machinery proteins are found at the periphery of the stacks (Tie et al., 2018).

Furthermore, on top of its classical roles in modification, sorting and transport of cargo, the Golgi apparatus has been assigned novel functions that seem to require an intact ribbon structure. For example, there is considerable evidence that it participates actively in cell signaling (Farhan et al., 2010; Chia et al., 2012; Luini and Parashuraman, 2016; Makhoul et al., 2018). The first signaling event, which is coupled to fragmentation of the Golgi ribbon was identified via the demonstration of a “Golgi checkpoint” regulating mitotic entry (Sütterlin et al., 2002; Colanzi et al., 2007). More recently, the coordinated trafficking and signaling functions of the Golgi apparatus have been implicated in complex cellular processes, such as cell migration, metabolism, and autophagy (Millarte and Farhan, 2012; Makhoul et al., 2018). Strikingly, the Golgi collaborates with the centrosome in providing a platform for the nucleation of MTs (Chabin-Brion et al., 2001; Efimov et al., 2007; Rivero et al., 2009), to support ribbon integrity, cell polarization and motility. Indeed, recent studies indicate that directional cell migration, which involves polarized delivery of lipids and proteins to the cell’s leading edge, depends on reorientation of both the centrosome and the Golgi ribbon, as well as an asymmetrical array of Golgi-nucleated MTs (Miller et al., 2009; Yadav et al., 2009; Hurtado et al., 2011). In sum, the discovery of these novel organelle functions raises questions regarding the division of labor between the compact and non-compact regions of the Golgi ribbon. In fact, in specific cell types, the non-compact zones amount to up to 50% of the total volume of the ribbon (Noske et al., 2008).

How are the stacks actually joined together? The prevailing view is based on stereoscopic EM analysis of serial sections in a variety of cell types (Rambourg and Clermont, 1997; Kepes et al., 2005) and fluorescence recovery after photobleaching (FRAP)-experiments demonstrating the continuity of the Golgi ribbon (Cole et al., 1996b; Puthenveedu et al., 2006). Accordingly, the cisternal stacks are thought to tether and fuse laterally, resulting in the formation of stable or transient tubular connections. Although it is commonly stated that such fusions only give rise to homotypic links between *cisternae* occupying equivalent positions in adjacent stacks (Figure 1), interconnections may be created between *cisternae* at different levels of neighboring stacks (Kepes et al., 2005). Of note, in many cell types the lateral tubular networks also appear to be in continuity with a tubular system at the *cis*-side of the Golgi ribbon (Kepes et al., 2005).

The joining of the Golgi stacks into a ribbon involves complex cellular machinery (Wei and Seemann, 2010; Mironov and Beznoussenko, 2011; Bechler et al., 2012; Rabouille and Linstedt, 2016; Huang and Wang, 2017). Both centrosome- and Golgi-nucleated MTs participate in this process, contributing to the central positioning or lateral linking of the stacks, respectively (Miller et al., 2009; Lowe, 2011; Yadav and Linstedt, 2011; Nakamura et al., 2012). More recently, an actin-based filament system that collaborates with MTs and Golgi-associated proteins, such as Cdc42, Rab1, and GRASP65 (Kodani et al., 2009; Hehnly et al., 2010; Copeland et al., 2016; Russo et al., 2016; Tang et al., 2016; Xing et al., 2016; Kage et al., 2017; Makhoul et al., 2019), has been implicated in ribbon formation (Egea et al., 2013; Gosavi and Gleeson, 2017). In addition, both membrane flow and cargo load influence the structure and function of the Golgi ribbon (Sengupta and Linstedt, 2011). Accordingly, its integrity depends on ongoing pre-, intra-, and post-Golgi membrane traffic (Yang et al., 2011; Climer et al., 2015; Blackburn et al., 2018; Makhoul et al., 2019) and blocking the transport of cargo-containing ER-to-Golgi carriers (Marra et al., 2007), or depletion of cargo receptors (Mitrovic et al., 2008), results in ribbon fragmentation. Finally, besides cell stress and apoptosis (Machamer, 2015) Golgi fragmentation is associated with various pathological conditions, including neurodegenerative disorders – such as amyotrophic lateral sclerosis (ALS), Alzheimer’s and Parkinson’s disease – and cancer. Notably, while the causative role of Golgi alterations in the progression of these diseases remains open, there are indications that they result from general effects on membrane traffic (Stieber et al., 1996; Fujita et al., 2006; Rendón et al., 2013; Joshi et al., 2015; Makhoul et al., 2018).

The identification of the roles of various transport machinery proteins – such as the GRASPs, golgins and regulatory GTPases – in ribbon formation is largely based on studies showing that their inhibition or depletion leads to Golgi fragmentation (de Figueiredo et al., 1998; Yadav and Linstedt, 2011; Goud et al., 2018). Two types of fragmentation can be distinguished: first, blocking MT- and dynein-dependent centralization of dynamic intermediate compartment (IC) elements and endosomes – as occurs in cells treated with nocodazole – gives rise to Golgi ministacks close to ERES. This situation is exemplified by knock-down of the dynein receptor golgin-160, or GMAP-210, a tethering protein (“golgin”) implicated in ER-Golgi trafficking at the level of the IC (Rios et al., 2004; Yadav et al., 2009; Roboti et al., 2015). In the second form of fragmentation, severing the Golgi ribbon – evidently due to local effects – leaves separated cisternal stacks residing at the cell center next to the centrosome.

In conclusion, the complex machineries implicated in the formation and maintenance of the Golgi ribbon, including local and global players, are difficult to reconcile with the currently popular model depicting narrow tubular connections between the Golgi stacks (Figure 1). This relatively simple model places the focus on the cisternal stacks as the basic structural and functional units of the ribbon, but does not adequately take into account the extensive tubular networks that – as mentioned earlier – represent an additional key feature of this organelle (Mollenhauer and Morré, 1998; Kepes et al., 2005). Moreover, several studies indicate that the ultrastructural organization of the non-compact zones within the ribbon is more complex than presented by the prevailing “tubular bridge model” (Thorne-Tjomsland et al., 1998; Ladinsky et al., 1999; Martínez-Martínez et al., 2017).

Therefore, based on the recently discovered spatial and functional connections between the membrane networks operating in ER-Golgi and endocytic trafficking (Marie et al., 2009, 2012; Bowen et al., 2017), we propose an alternative model for Golgi organization in vertebrate cells. According to this model these networks, which co-exist at the cell periphery and around the centrosome, also meet at the level of the Golgi ribbon, representing a permanent template that generates the transient Golgi stacks and simultaneously links them into a continuous structure. Unlike the “tubular bridge model”, this “linker compartments model” can clarify the tight coordination of the repositioning of the Golgi ribbon and the centrosome as a prerequisite for cell division and directed cell migration. It is also relevant for understanding the development of endomembranes and their rearrangements during cell differentiation. Furthermore, we discuss the implications of this model for enigmatic processes that take place at opposite sides of the Golgi stacks – such as MT nucleation and autophagy – as well as transport routes that pass through or circumvent the Golgi stacks.

But first, an introduction to the present terminology: In the following we refer to the two interconnected membrane systems defined by Rab1 and Rab11 as biosynthetic and endocytic networks, respectively, and their individual dynamic components as IC elements and recycling endosomes (REs). Based on their accumulation around the centrosome, the central domains of these networks have been previously designated as biosynthetic (BRC) and endocytic recycling compartments (ERC) (Maxfield and McGraw, 2004; Saraste and Goud, 2007). For simplicity, the IC elements and REs at non-compact zones of the Golgi ribbon have been dubbed here as “linker compartments”.

## Unlinking of the Golgi Ribbon During Cell Division and Migration: Two Alternative Views

**FIGURE 2:**
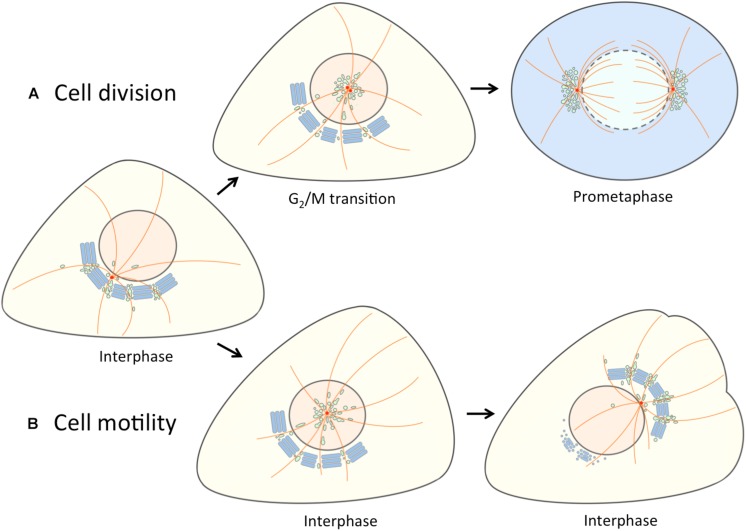
Separation of the linker compartments from the Golgi ribbon provides a landmark for the onset of mitosis and cell motility. At interphase the linker compartments, indicated with a single color (green) reside at the non-compact regions of the Golgi ribbon. **(A)** At late G2, the repositioning of the duplicated centrosomes is accompanied by the detachment of the linker compartments from the Golgi ribbon and their movement to the cell center along the radial array of centrosome-nucleated MTs (orange). As cells enter mitosis, the pericentrosomal compartments – BRC and ERC – expand and divide as the centrosomes mature, separate and move to form the spindle poles. At prometaphase, when the nuclear membrane breaks down, disassembly of the Golgi stacks (blue) gives rise to a vesicular “Golgi haze”, which together with the permanent compartments at the spindle poles contributes to the reassembly of the Golgi ribbon as cells exit mitosis (not shown). **(B)** The repolarization of the Golgi apparatus in motile cells is initiated by similar detachment of the linker compartments during the fragmentation of the Golgi ribbon. In this case, however, the joint reorientation of these compartments with the centrosome sets the stage for simultaneous reformation of the stacks and the Golgi ribbon on the other side of the nucleus facing the cell’s leading edge.

Despite its complex organization the Golgi apparatus is capable of rapidly changing its shape and cellular location under different physiological conditions. Typically, such dynamic alterations coincide with the repositioning of the centrosome and the unlinking of the Golgi ribbon (Rios and Bornens, 2003; Sütterlin and Colanzi, 2010). These events are necessary, for instance, for equal partitioning of this single-copy organelle during cell division and its reorientation toward the lamellipodium during cell migration. In the following, we discuss these two cellular processes in light of the commonly accepted “tubular bridge model” of the Golgi ribbon (Figure 1) and the “linker compartments model” proposed here (see Figure 2). Golgi rearrangements are also an integral part of cell differentiation, taking place, for example, during the formation of neuronal extensions and the polarization of epithelial cells (see below; Figure 5).

The best-characterized process of physiological Golgi fragmentation takes place as cells prepare for mitosis. At the late G2 stage of the cell cycle the mammalian Golgi ribbon breaks down into individual stacks due to activation of the membrane fission protein CtBP1/BARS (Hidalgo-Carcedo et al., 2004; Colanzi et al., 2007), and phosphorylation of the two tethering proteins GRASP65 and GRASP55 (Sütterlin et al., 2002; Yoshimura et al., 2005; Feinstein and Linstedt, 2007, 2008; Duran et al., 2008; Cervigni et al., 2015). However, the precise roles of these two factors in this process remain incompletely understood (Ayala and Colanzi, 2017). For example, whereas the function of the GRASPs in homotypic tethering of membranes (via *trans*-oligomerization) has been extensively characterized (Rabouille and Linstedt, 2016), the mechanism of CtBP1/BARS activation remains unknown. Nevertheless, evidently as a consequence of the joint action of the GRASPs and CtBP1/BARS, the initially asymmetric juxtanuclear Golgi stacks end up circling the nucleus as the cells reach prophase, coinciding with the separation of the centrosomes and initiation of formation of the mitotic spindle (Shorter and Warren, 2002; Wei and Seemann, 2017). If Golgi fragmentation is blocked – for example, by inhibiting CtBP1/BARS activation or the phosphorylation of one of the GRASPs – the progression of cells from G2 to prophase is delayed. This regulatory event of the cell cycle is referred to as the Golgi checkpoint (Sütterlin et al., 2002; Colanzi et al., 2007). A similar controlled unlinking process occurs during mitotic entry in *Drosophila* S2 cells, despite the fact that the fly Golgi is not a ribbon, but exists as pairs of stacks. Notably, however, in this case the linking or unlinking of the stacks does not involve the single *Drosophila* GRASP homolog (dGRASP), but is mediated by the Golgi-associated actin cytoskeleton (Kondylis et al., 2007).

Directed migration of fibroblasts is also accompanied by unlinking of the Golgi ribbon, followed by its subsequent relocation to the side of the nucleus facing the leading edge (Kupfer et al., 1982). This process ensures polarized delivery of membrane constituents – lipids and specific proteins, such as integrins – to the leading edge, thereby supporting cell polarization and directed motility (Bisel et al., 2008; Millarte and Farhan, 2012). Besides contributing to linking of the stacks, Golgi-nucleated MTs establish an asymmetric array of filaments, providing tracks for polarized trafficking to the lamellipodium (Efimov et al., 2007; Miller et al., 2009; Rivero et al., 2009). While Golgi and the centrosome are thought to part company as cells enter mitosis (Champion et al., 2017), Golgi relocation during cell migration is intimately coupled to repositioning of the centrosome (Sütterlin and Colanzi, 2010; Hurtado et al., 2011). In fact, unlinking of the Golgi stacks appears to be a prerequisite for repositioning of the centrosome (Preisinger et al., 2004; Bisel et al., 2008; Millarte and Farhan, 2012). Namely, as in mitosis, this process depends on phosphorylation of GRASP65, and inhibition of this modification – for example, using non-phosphorylatable mutants – blocks centrosome positioning and cell polarization (Bisel et al., 2008). Cell migration is also regulated by GM130, which associates with IC/*cis*-Golgi membranes via GRASP65 (Preisinger et al., 2004; Saraste and Marie, 2018). GM130 could affect cell polarization and migration via multiple mechanisms (Sütterlin and Colanzi, 2010). One could involve interaction with the Rho family GTPase Cdc42, a key regulator of cell polarization (Etienne-Manneville, 2004; Kodani et al., 2009; Baschieri et al., 2014; see below). Another possible role of GM130 in cell migration could depend on its function in Golgi nucleation of MTs, which provide tracks for transport to the lamellipodium (Rivero et al., 2009). Furthermore, GM130 provides a scaffold for the activation of kinases (YSK1 and MST4) that regulate cell migration (Preisinger et al., 2004).

In summary, the two types of events leading to Golgi fragmentation, taking place at G2/M transition or during cell migration, are at least partly regulated by different signaling pathways (Millarte and Farhan, 2012; Ayala and Colanzi, 2017). Also, the extent of Golgi disassembly differs in these two cases. During mitosis the Golgi undergoes a multi-step disassembly process, which results in the appearance of two components: tubulovesicular membrane clusters concentrating at the spindle poles and a vesicular Golgi haze (Marie et al., 2012; Wei and Seemann, 2017). By contrast, Golgi reorganization during cell migration seems to be less dramatic, possibly limited to unlinking of the ribbon and partial breakdown of the Golgi stacks (Bisel et al., 2008). Interestingly, GRASP65 is phosphorylated at the same site (Ser 277) by ERK or JNK2 during cell migration and mitotic entry, respectively, indicating that Golgi fragmentation during these cellular events shares similar mechanisms. Based on the “tubular bridge model”, a commonly held view is that the molecular changes in both cases initially trigger the severing of the tubular connections between the relatively stable cisternal stacks, resulting in the unlinking of the Golgi ribbon. As a consequence, the individual Golgi stacks are thought to be released and even become mobile, allowing their repositioning.

### A New View of the Golgi Ribbon

Our new model regarding the functional organization of the Golgi ribbon and its behavior at the onset of mitosis and during cell motility (Figures 2, 4) embodies the idea that the non-compact regions are structurally and functionally more complex than proposed by the “tubular bridge model”. It is based on the discovery of permanent connections between the IC and the endocytic recycling system and the anchoring of the two networks at the centrosome (Marie et al., 2009, 2012; Bowen et al., 2017; Saraste and Marie, 2018). Indeed, a direct link between the pericentrosomal IC elements and recycling endosomes (REs) – defined by the GTPases Rab1 and Rab11, respectively – persists when the Golgi stacks are disassembled by Brefeldin A (BFA; Marie et al., 2009), a reversible inhibitor that dissociates specific protein coats (COPI, clathrin) from membranes and has been extensively used to study endomembrane organization and protein transport in different cell types (Klausner et al., 1992; Prydz et al., 1992; Marie et al., 2008; Robinson et al., 2015). Here, we propose that – in addition to meeting at the cell periphery and around the centrosome – the central IC elements and REs also co-exist at the non-compact regions of the ribbon (Figure 2). Here they co-operate in the biogenesis of Golgi *cisternae* and consequently act as “linker compartments” that connect the stacks (Figure 4) in a process which is expected to be more dynamic than the one depicted in the “tubular bridge model”.

The alternative model is supported by EM tomographic studies of both cultured cells and tissues, providing high-resolution data on the non-compact regions of the Golgi ribbon (Ladinsky et al., 1999; Marsh et al., 2001; Martínez-Martínez et al., 2017). Ultrastructural analysis shows that these linker regions – besides displaying apparently stable tubular or saccular connections between the neighboring Golgi stacks – are characterized by large openings. Notably, these “wells” are filled with pleiomorphic structures resembling IC elements and endosomes, as well as tubules and coated or non-coated vesicles (Ladinsky et al., 1999). In pancreatic β-cells, where MTs are predominantly nucleated at the Golgi (Zhu et al., 2015), these filaments typically associate with *cis*-Golgi *cisternae* and endo-lysosomal compartments in the vicinity of the Golgi ribbon (Marsh et al., 2001). In addition, MTs can be seen passing through the non-compact zones (Marsh et al., 2001; Martínez-Martínez et al., 2017). Collectively, the above features support the conclusion that the non-compact regions represent structurally complex sites for dynamic transport events, rather than consisting solely of narrow tubular connections between the stacks.

Furthermore, it has been recognized for quite some time that a typical feature of the Golgi apparatus in many cell types is the presence of extensive tubular networks (Mollenhauer and Morré, 1998). Indeed, such networks represent a conserved aspect of Golgi structure, being present in animals, plants, and fungi, and – corresponding to roughly half of the total Golgi membrane – can also be expected to play an important role in Golgi function. Importantly, besides the *cis*- and *trans*-aspects of the stacks, they are also found on their lateral sides, contributing in vertebrate cells to the establishment of the non-compact regions of the ribbon. These regions also include saccular elements and display continuity with forming secretory granules. Notably, Mollenhauer and Morré proposed that the tubular networks represent the permanent components of the Golgi ribbon, whereas the Golgi stacks – based on the cisternal progression model – were expected to undergo continuous turnover (Mollenhauer and Morré, 1998).

Figure 2 shows the application of our alternative “linker compartments model” in the context of Golgi rearrangements taking place during cell division and motility. Experimental support for the mitosis model (Figure 2A) was obtained by live cell imaging of cells expressing the IC marker GFP-Rab1. At late G2, jointly with the movement of the duplicated centrosome to the cell center, a pool of IC membranes detaches from the Golgi ribbon. At prophase, this compartment (designated as BRC) – together with the Rab11-positive ERC – first grows and then divides as the centrosomes separate, and finally moves together with the latter to the forming spindle poles (Marie et al., 2012; Figure 2A). Since the separation and expansion of these pericentrosomal compartments coincide with the unlinking of the Golgi ribbon, we proposed that they are derived from its non-compact regions (Marie et al., 2012). How do the “linker compartments” pile up around the centrosome? A simple scenario is that as a consequence of the unlinking of the Golgi stacks and the release of these compartments from the ribbon – for example, due to membrane untethering and/or cytoskeletal rearrangements – they are free to move toward the centrosome in a dynein-dependent fashion, using the radiating centrosomal MTs as tracks (Figure 2A).

**FIGURE 3:**
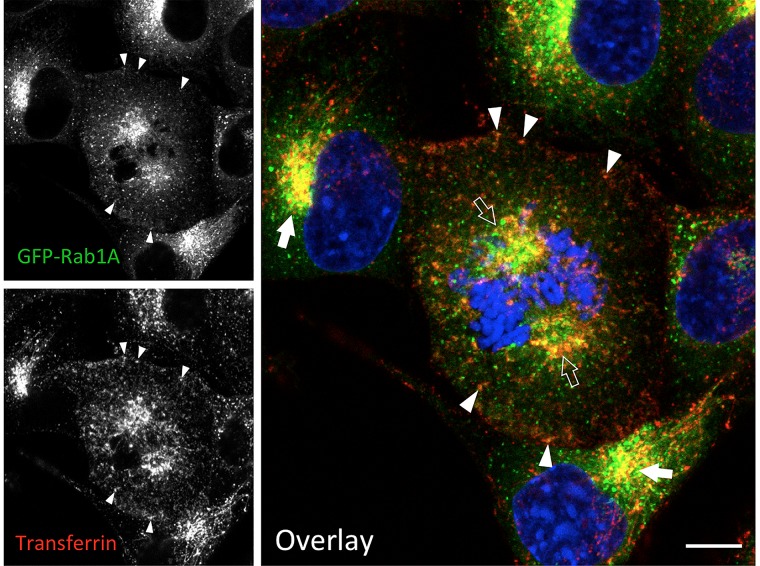
IC elements and REs persist and co-localize during mitosis. Normal rat kidney (NRK) cells stably expressing green fluorescent protein (GFP)-coupled Rab1 as a marker for the IC were labeled with fluorescent transferrin during a 1 h uptake to visualize the endosomal recycling system. At the same time, the cells were exposed to BFA, which disassembles the Golgi stacks, but does not affect mitotic entry or progression. Note the co-localization of the IC elements and REs at the spindle poles of a cell that has reached prometaphase (open arrows), as well as in the pericentrosomal area of interphase cells (arrows) and. In addition, co-localization of the two markers is observed at peripheral sites (arrowheads). The interphase nuclei and mitotic chromosomes are stained with DAPI. Bar = 5 μm (see also Marie et al., 2012; Takatsu et al., 2013).

Besides Rab11 (Marie et al., 2012; Hehnly and Doxsey, 2014), the pericentrosomal ERC at the spindle poles can be visualized via endocytosed transferrin, or antibodies against its receptor (Takatsu et al., 2013; Figure 3). The BRC also contains the Rab1 effectors GM130 and p115 (Seemann et al., 2002; Radulescu et al., 2011), as well as GRASP65, which provides a membrane anchor for GM130 (Marie et al., 2012). Based on their proposed function in the biogenesis and maintenance of the Golgi stacks (Saraste and Marie, 2018), the linker compartments dynamically interact with the stacks during interface, as well as with the vesicular Golgi haze during mitosis (Marie et al., 2012). Therefore, Golgi enzymes may also be found at the spindle poles, as a consequence of their missorting due to overexpression and/or tagging.

**FIGURE 4:**
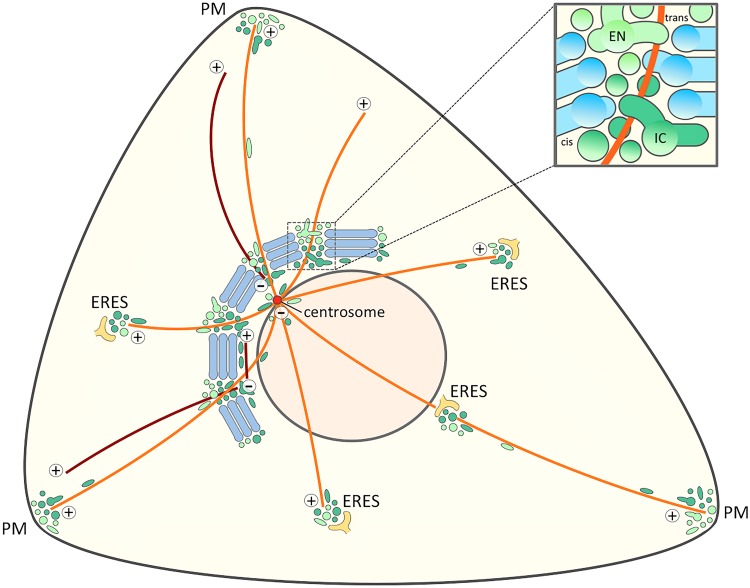
Signaling and trafficking along a cell periphery – Golgi – centrosome axis. The proposed joint operation of the IC elements (dark green) and REs (light green) as linker compartments in the Golgi ribbon sets the stage for MT-dependent pathways that connect the cell periphery with the Golgi and the centrosome at the cell center. Besides providing a possible axis for cell signaling this direct connection opens up for transport pathways that bypass the Golgi stacks. Furthermore, the existence of a direct link between IC elements and the cell periphery (Sannerud et al., 2006) raises the possibility that the IC elements and endosomes also meet at ERES. For simplicity, a structure consisting of five stacks displaying uniform *cis-trans* polarity is shown, while in reality the Golgi ribbon is a twisted, basket-shaped structure in the perinuclear area of a fibroblastic cell. The blow-up illustrates a non-compact region of the Golgi ribbon. The linker compartments derived from the central domains of biosynthetic (IC) and endocytic (EN) networks are schematically depicted as separate structures, although they are expected to establish tubular and saccular continuities between the neighboring stacks (blue). The centrosomal and non-centrosomal (Golgi-nucleated) MTs with plus-minus polarity are indicated in orange and brown color, respectively.

The present model suggests that the Golgi ribbon consists of two main domains with distinct properties (Figures 2, 4). The non-compact linker regions are considered as the permanent part of the ribbon, which function in the formation of the transient Golgi stacks. As discussed above, the linker compartments are expected to actively communicate with the stacks via vesicular or tubular trafficking. In addition, they can establish more stable connections, allowing communication between neighboring Golgi stacks. This two-component model is in accordance with results suggesting that different parts of the Golgi employ different inheritance strategies (Wei and Seemann, 2009). Thus, the vesicular Golgi haze – evidently together with linker compartments at the cell periphery – can generate transport-competent Golgi stacks, while a spindle-associated component is required for post-mitotic ribbon formation. Detailed studies of the Golgi reassembly process during mitotic exit can address the validity of this two-domain model. Interestingly, during cytokinesis the reforming Golgi elements in the daughter cells first organize into two unequal membrane clusters at the two sides of the nuclei. The smaller Golgi cluster (“twin Golgi”), localized near the intercellular bridge, then moves to the opposite side of the nucleus to join the larger pericentrosomal Golgi cluster during reformation of the interphase Golgi ribbon (Gaietta et al., 2006; Marie et al., 2012).

The model regarding the role of the pericentrosomal compartments in Golgi repositioning during cell migration (Figure 2B) is also based on live imaging of GFP-Rab1 (Marie et al., 2009). Similarly as during G2/M transition, the IC membranes are relocated with the centrosome to the cell center as the cell starts to move. Subsequently, the Rab1-containing IC/*cis*-Golgi membranes are transferred to the opposite side of the nucleus, apparently utilizing the centralized pericentrosomal compartment as a way station. Finally, due to the function of the linker compartments in reformation of the Golgi stacks, the reoriented Golgi ribbon – simply based on spatial constraints – is positioned at a distance from the centrosome to face the leading edge (Figure 2B).

As mentioned earlier, treatment of cells with BFA results in breakdown of the Golgi stacks and accumulation of the linker compartments around the centrosome (Marie et al., 2009), creating a situation very similar to that seen during mitotic onset and cell motility (Figure 2). Therefore, it does not come as a total surprise that BFA can “rescue” both an experimentally induced block in mitotic entry (Sütterlin et al., 2002; Feinstein and Linstedt, 2007; Cervigni et al., 2015), and centrosome reorientation in motile cells where ribbon fragmentation has been experimentally inhibited (Bisel et al., 2008). Of note, the linker compartments maintain their close connection during mitosis (Marie et al., 2012; Takatsu et al., 2013; Hehnly and Doxsey, 2014; Figure 3), as well as during cell migration (Dale et al., in preparation). Moreover, wound-healing assays reveal that cell motility is not inhibited, but rather enhanced, during the first hours of BFA treatment (Dale et al., in preparation). Furthermore, the ability of BFA to rescue centrosome positioning as a prerequisite to cell migration revealed how critically dependent this process is on the unlinking of the Golgi ribbon (Bisel et al., 2008). Based on the “tubular bridge model” it looked as if the extensive Golgi ribbon would somehow be able to mechanically or sterically inhibit centrosome motility. Simultaneous repositioning of the linker compartments and the centrosome (Figure 2) may solve this puzzle and explain the tight coordination of these processes, which may both involve the master of cell polarization, the GTPase Cdc42 (see below).

Finally, these considerations set the stage for a new view of Golgi positioning. According to one popular view the Golgi ribbon is first fragmented, whereafter the individual stacks are free to move across the cytoplasm to find their new location. Alternatively, resident Golgi enzymes may redistribute to the ER and organelle repositioning involves *de novo* assembly of Golgi stacks at ERES, resulting in ribbon formation at a new site. As a trade-off, the new model emphasizes a novel role of the linker compartments in defining Golgi repositioning, either at the distal side of the daughter nuclei at telophase (Figure 2A), or facing the leading edge of a motile cell (Figure 2B). We propose that in both situations the *cis/medial*- and *trans*-Golgi residents are redistributed to the permanent IC and endosomal networks, respectively, and relocate together with these dynamic elements, resulting in reformation of the Golgi stacks at new locations. The difference is that during mitosis Golgi enzymes are further distributed to the vesicular Golgi haze which, however, still communicates with the compartments at the spindle poles (Figure 2A; Marie et al., 2012).

## Spatial Aspects of Trafficking and Signaling

The localization of endosomes and IC elements at the cell center is based on their dynein-dependent movements along MT tracks (Burkhardt et al., 1997; Presley et al., 1997; Horgan et al., 2010; Granger et al., 2014). The positioning of these compartments at the non-compact zones of the Golgi ribbon, at a distance from the centrosome (Figure 4), could be based on simple spatial constraints, created by their centralization and function in the formation of the sizeable Golgi stacks. Alternatively, it could be influenced by their association with actin filaments, mutual adhesion – for example, the establishment of membrane contact sites – or the opposing forces generated by MT motors. Indeed, both the IC elements and REs (containing Rab1 and Rab11, respectively) are capable of moving bidirectionally along MTs. As a consequence, they are also found at the cell periphery (Figure 4); for example, in the protrusions or lamellipodia of migrating fibroblasts, and neuronal growth cones (Hattula et al., 2006; Sannerud et al., 2006; Eva et al., 2010; Matsuzaki et al., 2011; Takahashi et al., 2012). Unexpectedly, the well-established IC/*cis*-Golgi proteins Rab1, Arf1 and GBF1 – the GTP exchange factor of the latter – have been shown to act in endocytic trafficking (Gupta et al., 2009; Mukhopadhyay et al., 2011; Kaczmarek et al., 2017). Arf1 regulates a constitutive clathrin-independent endocytic pathway, which is also mediated by Cdc42, and plays a major role in membrane turnover at the leading edge of migrating cells. Strikingly, the protein profile of the clathrin-independent carriers (CLICs) operating in this pathway includes also the IC proteins Rab1, Sec22b and p58/ERGIC-53 (Howes et al., 2010).

Together with their enrollment as linker compartments in the Golgi ribbon these considerations provide a new view on the spatial organization of the MT-based early biosynthetic (IC) and endocytic membrane networks. The emerging cell surface – Golgi ribbon – centrosome axis (Figure 4) can provide an explanation for the striking operation of the same transport machineries both at the ER-Golgi boundary and the cell periphery. In the following, we also address the implications of this novel axis for signaling events that regulate the onset of mitosis or cell migration. Furthermore, by shifting the main focus away from the *cisternal* Golgi stacks, the model is relevant for considering the localization and function of machinery proteins implicated in ribbon formation, such as the GRASPs, as well as Golgi-independent pathways of protein and lipid trafficking.

### Signaling at the Golgi Checkpoint

As discussed above, the fragmentation of the Golgi ribbon at G2 is linked to cell cycle control mechanisms, coinciding with the “Golgi checkpoint” that regulates mitotic entry (Sütterlin et al., 2002; Hidalgo-Carcedo et al., 2004; Yoshimura et al., 2005; Colanzi et al., 2007). Based on the prevailing model of the ribbon (Figure 1), the general idea is that this control station monitors the successful splitting of the continuous Golgi ribbon into individual stacks. Thus, despite the fact that severing the tubular connections between the stacks marks only the beginning of a multi-step Golgi disassembly process, the consensus is that the checkpoint oversees organelle inheritance (Wang and Seemann, 2011; Ayala and Colanzi, 2017). Some of the signaling events that link Golgi integrity to mitotic entry have recently been identified. Accordingly, ribbon fragmentation at late G2 leads to the activation of a Golgi-localized Src kinase, which phosphorylates another key kinase, Aurora A, resulting in its activation and recruitment to the centrosome (Persico et al., 2010; Barretta et al., 2016). This event is a prerequisite for centrosome maturation, including expansion of the pericentrosomal material, which affects MT nucleation and formation of the mitotic spindle (Wei et al., 2015; Barretta et al., 2016). Importantly, the recruitment of activated Aurora A to the centrosome culminates in the activation of Cdk1, the master kinase that sets mitosis in motion (Champion et al., 2017).

What is the mechanism that couples Golgi unlinking to centrosome maturation? How does the apparently *trans*-Golgi/TGN-localized Src kinase come in contact with Aurora A at the centrosome? The proposed behavior of the linker compartments at the onset of mitosis (Marie et al., 2012; Figure 2A) may provide an answer. Namely, their detachment from the Golgi ribbon at late G2 and movement to the pericentrosomal region may constitute the pathway that mediates the interaction of the two kinases and the recruitment of activated Aurora A to the centrosome (Barretta et al., 2016). Indeed, similar relocation of TGN proteins to the pericentrosomal area takes place when the Golgi stacks are disassembled by BFA (Reaves and Banting, 1992; Molloy et al., 1994). This Golgi ribbon-centrosome axis could also act in the transfer of other key proteins that regulate mitotic entry, such as cyclin B2, the partner of Cdk1 (Jackman et al., 1995) and the phosphatase Cdc25C, an activator of the cyclin B2/Cdk1 complex (Noll et al., 2006). In general, the pericentrosomal accumulation of the IC elements and REs (Marie et al., 2012) could play an important role in the maturation (at G2) and separation (at prophase) of centrosomes, as well as formation of the MT-based mitotic spindle (Hehnly and Doxsey, 2014; Wei et al., 2015; Ibar and Glavic, 2017).

The models in Figures 2A, 4 also provide a new perspective to consider the nature of the Golgi checkpoint operating at the G2/M transition. Instead of overseeing the unlinking of the presumably transient Golgi stacks, this control station could monitor the state of the two permanent membrane systems – the biosynthetic (IC) and endocytic networks – meeting at the non-compact zones of the Golgi ribbon. In case they are found ready for accurate partitioning (Marie et al., 2012), and competent to carry out their mitotic roles, the linker compartments detach from the Golgi ribbon, and relocate to the centrosome. However, if damage is detected (or something is missing), their separation is arrested, and entry into mitosis is delayed. Accordingly, the check-point can control both cell cycle progression and organelle inheritance. This scenario is also compatible with the striking finding that the progression of cells through mitosis is not affected by the presence of BFA (Seemann et al., 2002; Nizak et al., 2004; Marie et al., 2012; Figure 3). Although BFA disassembles the Golgi stacks, it allows the linker compartments to detach, partition properly in parallel with centrosome separation, and evidently also support basic trafficking and signaling events that take place during mitosis. Thus, besides their initial unlinking, the subsequent mitotic fate of the cisternal Golgi stacks is a secondary issue. Recently, experimental filling of the Golgi lumen with DAB precipitate was shown to allow mitotic entry, but inhibit the disassembly of the Golgi stacks, resulting in mitotic arrest at the spindle assembly checkpoint (SAC; Guizzunti and Seemann, 2016). An alternative explanation is that the function of the linker compartments is also affected by this treatment, as indicated by the inability of the centrosomes to separate properly. Thus, the ensuing damage to these compartments, rather than that of the Golgi stacks, is the reason for SAC activation.

As cells prepare for division, they change both their shape and internal architecture. At late G2, based on the initial disassembly of integrin-based focal adhesions (FAs) they begin to round up. This dramatic alteration in cell shape is transmitted via the cortical actin meshwork and the radiating MT system to the cell center, resulting in the positioning of the centrosome to the geometric center of the cell and redistribution organelles, such as the Golgi apparatus (Champion et al., 2017). Notably, cell cycle progression is also controlled from the distance, as specific FA components move from the cell surface to the centrosome, where they interact with Aurora A, thereby influencing centrosome maturation and mitotic entry (Pugacheva and Golemis, 2006). Such a complex control of Aurora A activation may involve the trafficking and signaling pathways proposed in Figure 4, which not only connect the centrosome with the Golgi ribbon, but also with FAs at the cell periphery. The localization of GBF1 and Arf1 to adhesion sites at the leading edge (Mazaki et al., 2012; Schlienger et al., 2015; Busby et al., 2017) and the proposed roles of Rab1 and Rab11 in integrin trafficking and cell adhesion (Wang et al., 2010; Paul et al., 2015) are in accordance with this possibility.

### Coordinating Golgi and Centrosome Positioning

Another possible example of cross-talk between the cell surface, non-compact zones of the Golgi ribbon and the centrosome (Figure 4) is provided by the function of the master regulator of cell polarity – the GTPase Cdc42 of the Rho family – during cell migration (Etienne-Manneville, 2004). Activation of Cdc42 at the leading edge of migrating cells triggers actin polymerization, promoting the formation of cellular protrusions and stabilization and anchoring of the plus-ends of MTs at the actin-based cortical filament meshwork of the lamellipodium. Accordingly, a plasma membrane-associated pool of Cdc42 directs the relocation of the centrosome between the nucleus and the leading edge in a MT- and dynein-dependent process that is intimately coupled to Golgi repositioning. Due to reorientation of the MT network, post-Golgi and RE carriers are directed to the lamellipodium, setting the stage for cell polarization and migration.

Another pool of Cdc42 is present in the Golgi region where it interacts with COPI coats and GM130, suggesting that it associates – at least partly – with IC/*cis*-Golgi membranes (Erickson et al., 1996; Wu et al., 2000; Kodani et al., 2009; Baschieri et al., 2014). In addition, EM has shown the predominant localization of Cdc42 to tubulovesicular membranes at the lateral sides of the Golgi stacks (Luna et al., 2002), in line with the possibility that it also associates with the linker compartments at the non-compact zones of the Golgi ribbon. Indeed, Cdc42 can be recruited from this central pool to the cell surface in an MT- and Arf6-dependent manner, indicating its presence in the REs (Osmani et al., 2010; Baschieri et al., 2014; Farhan and Hsu, 2016). Notably, it also functions in dynein-dependent endosome-to-Golgi trafficking (Hehnly et al., 2009), as well as dynein recruitment to COPI-coated ER-to-Golgi (or intra-Golgi) carriers, indicating a role in Golgi positioning (Hehnly et al., 2010). Therefore, it is tempting to speculate that Cdc42 also regulates the dynein-based pericentrosomal accumulation of the linker compartments during cell migration (Figure 2B). Furthermore, the concerted actions of the peripheral and central pools of Cdc42 could explain the tight coupling of centrosome and Golgi re-positioning during this process. Additional effects of Cdc42 on actin dynamics (Luna et al., 2002; Hehnly et al., 2010), MT nucleation, bidirectional trafficking and/or the kinetics of anterograde transport at the Golgi ribbon (Park et al., 2015) could also contribute to Golgi repositioning and polarized delivery of membrane to the leading edge of migrating cells (Farhan and Hsu, 2016).

Is its possible that Cdc42 cycles between the cell periphery and the non-compact zones of the Golgi ribbon (Figure 4)? Namely, other GTPases of the Rho-family have been suggested to be transferred from the PM to the ERC (Bouchet et al., 2018). Cdc42 could employ the clathrin-independent endocytic pathway regulated by GBF1 and Arf1. Whatever the precise route, such cycling could explain how the PM pool of Cdc42 can regulate centrosome organization (Kodani and Sütterlin, 2008; Kodani et al., 2009; Herrington et al., 2017).

### A New Role for GRASPs?

In addition to tethering Golgi *cisternae* into stacks via their ability to *trans*-oligomerize, the two mammalian GRASP proteins, GRASP55 and GRASP65, have been implicated as key players in the process that links the Golgi stacks into a ribbon (Puthenveedu et al., 2006; Feinstein and Linstedt, 2008; Jarvela and Linstedt, 2014; Veenendaal et al., 2014; Rabouille and Linstedt, 2016; Bekier et al., 2017; Huang and Wang, 2017). As discussed above, the strongest evidence for the latter role comes from the demonstration that phosphorylation of the GRASPs is required for Golgi fragmentation and entry of cells into mitosis (Ayala and Colanzi, 2017).

Although GRASP65 and GRASP55 are generally referred to as *cis*- and *medial*/*trans*-Golgi proteins, respectively, and there is evidence suggesting that they function separately to link *cisternae* at the *cis*- and *trans*-sides of the stacks (Jarvela and Linstedt, 2014; Rabouille and Linstedt, 2016), their ultrastructural localizations within the Golgi ribbon have not been firmly established. Interestingly, however, besides being found predominantly at the *cis*- and lateral sides of the Golgi stacks, the single GRASP (dGRASP) in *Drosophila* S2 cells has been localized by EM to pleiomorphic tubulo-vesicular elements at the ER-Golgi boundary (Kondylis et al., 2005). More recently, super-resolution microscopy placed both mammalian GRASPs to the *cis*-side of Golgi ministacks, displaying a localization similar to that of Rab1 (Tie et al., 2018). Moreover, both GRASPs co-localize extensively with Rab1 (Marie et al., 2012; own unpublished data), and there is also previous evidence suggesting IC localization of GRASP65 (Marra et al., 2001).

Notably, at metaphase – following disassembly of Golgi stacks – GRASP65 is found at the spindle poles (Marie et al., 2012). Therefore, the model of Golgi fragmentation during G2/M transition (Figure 2B) opens the possibility that GRASP65 – like Rab1 – is present at the linker regions of the Golgi ribbon. This localization would be compatible with its proposed dual role in assembling the cisternal stacks and linking the Golgi ribbon (Rabouille and Linstedt, 2016; Huang and Wang, 2017), and its function in anterograde trafficking (D’Angelo et al., 2009). It might also explain why GRASP65 depletion accelerates cell surface delivery of certain proteins (such as APP, integrin and CD8), affects protein glycosylation and results in missorting of cathepsin D (Xiang et al., 2013; Bekier et al., 2017; Huang and Wang, 2017). Furthermore, one of the GRASPs might even participate in the tethering of the linker compartments at the non-compact zones. Namely, despite lacking Golgi stacks the yeast *S. cerevisiae* contains a GRASP ortholog Grh1 (Behnia et al., 2007). Moreover, it seems likely that the tubulovesicular networks that constitute the yeast secretory pathway correspond to the biosynthetic and endocytic networks that meet at the non-compact regions of the mammalian Golgi ribbon (Marie et al., 2008; Jackson, 2009; Saraste and Marie, 2018).

Finally, the new model on the spatial organization and dynamics of the biosynthetic (IC) and endocytic networks proposed in Figure 4 could also explain the findings showing that the mammalian and fly GRASPs can exert their functions not only at the Golgi, but also close to ERES (Kondylis et al., 2005; Kim et al., 2016), or even at the cell periphery (Schotman et al., 2008).

### Bypassing the Golgi Stacks

Another argument for placing the GRASPs at the non-compact zones of the Golgi ribbon is their participation in Golgi-independent secretory pathways that an increasing number of proteins employ during their intracellular trafficking (Prydz et al., 2013; Gee et al., 2018). Generally, a set of transmembrane proteins – including receptors, ion channels and adhesion proteins – entering the secretory pathway at the ER can reach the cell surface without passing through the Golgi stacks (Martin et al., 2001; Baldwin and Ostergaard, 2002; Yoo et al., 2002; Marie et al., 2008; Hanus et al., 2016). In addition, certain cytoplasmic proteins lacking a signal sequence for ER translocation can reach the extracellular space by crossing the cell membrane directly, or after inclusion into membrane-bound organelles, which fuse with the PM (Dimou and Nickel, 2018). In a variety of organisms, both types of unconventional secretion may involve GRASPs, either for the departure of proteins from the classical secretory route, or their direct inclusion into transport carriers that are related to autophagosomes (Kinseth et al., 2007; Schotman et al., 2008; Dupont et al., 2011; Manjithaya and Subramani, 2011; Kortvely et al., 2016; Zhao et al., 2019). The diversion of the cystic fibrosis-related chloride channel (CFTR) into a Golgi bypass route takes advantage of components of the autophagic machinery and is stimulated by ER stress (Gee et al., 2011, 2018; Noh et al., 2018). Starvation of yeast cells triggers secretion via CUPS (compartment for unconventional protein secretion), a membrane structure formed by ER-Golgi system with contributions from the endosomal pathway (Cruz-Garcia et al., 2018). Notably, there is a close relationship between GRASP-dependent autophagy and GRASP-dependent unconventional secretion (Subramani and Malhotra, 2013). Some unconventional proteins pass via REs, where Rab11A regulates the secretion of e.g., α-synuclein (Liu et al., 2009; Chutna et al., 2014).

Also concerning anterograde trafficking, proteoglycan protein cores that bypass the Golgi apparatus when Golgi passage is inhibited will appear at the PM without polymerized glycosaminoglycan chains. When the proteoglycan serglycin was expressed in epithelial MDCK cells and Golgi bypass was induced by BFA, apical targeting was maintained, indicating that polarized sorting had already taken place prior to Golgi entry, presumably in the IC (Tveit et al., 2009).

Furthermore, the model in Figure 4 could help to understand the enigmatic itineraries taken by protein toxins during their retrograde transport from the cell surface to the ER. However, while different ER-destined toxins – such as ricin, Shiga, pertussis, and cholera toxins – all engage endogenous cell components to move retrogradely, they do not depend entirely on the same mechanisms at every step of the way (Sandvig et al., 2013). For example, while Shiga and cholera toxins (B subunit) move via REs to the Golgi apparatus in a retromer- or clathrin/AP-1-dependent fashion, respectively, ricin moves from early endosomes to the Golgi apparatus independently of Rab11 and clathrin (Mallard et al., 1998; Iversen et al., 2001; Matsudaira et al., 2015). Ricin may even reach the ER without encountering Golgi enzymes, most likely by an alternative retrograde Golgi bypass mechanism (Llorente et al., 2003). Since the focus on toxin entry to the Golgi apparatus has been at the *trans*-side, a number of studies have employed recombinant toxins with a sulfation site to monitor retrograde Golgi passage. Based on its permanent character (Saraste and Marie, 2018), it would be logical to assume that the IC is an obligate way station between the Golgi *cisternae* and the ER; however, this compartment has generally not been addressed in such studies. Nevertheless, the view that toxins destined for the ER pass through the IC is strengthened, since deletion of Rab1A or Rab1B, or expression of Rab1 mutants, impairs ricin toxicity (Simpson et al., 1995; Bassik et al., 2013). Also, ricin intoxicates mutant CHO cells (END4), where a typical Golgi apparatus disappears, but the IC seems to remain intact (Bau and Draper, 1993). Knockdown of intracellular phospholipase A1 γ (iPLA1γ), which is localized to the IC/*cis*-Golgi, blocked the ER delivery of cholera toxin B subunit, but not of Shiga toxin (Morikawa et al., 2009). An intact Golgi ribbon is not required for retrograde toxin trafficking, since *Drosophila* cells, where the dispersed Golgi apparatus existing as individual or pairs of stacks (Kondylis et al., 2007) are also sensitive to ricin (Pawar et al., 2011).

A further indication that molecules pass from RE to IC during their recycling is provided by studies of the cell surface heparan sulfate (HS) proteoglycan Glypican-1, which enters the endocytic pathway where the HS chains are trimmed down by glycosidases, before the protein scaffold recycles to the early secretory pathway to obtain novel HS chains (Mani et al., 2000).

## Golgi Remodeling by Differentiated Cells

**FIGURE 5:**
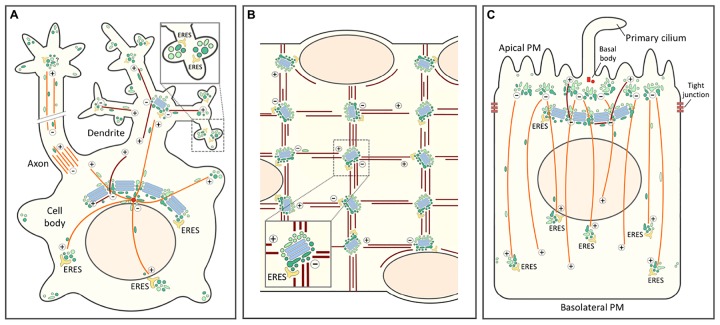
Role of the biosynthetic and endocytic networks in the organization of endomembranes and Golgi positioning in differentiated cell types. In all cells the centrosomal (or centrosome-derived) and non-centrosomal (Golgi-nucleated) MTs with plus-minus polarity are indicated by orange and brown color, respectively. The IC elements (dark green) and REs (light green) are also depicted by different colors. **(A)** Highly schematic model of a *neuron* with its cell body, axon and dendritic tree. Golgi stacks (blue) are present in the cell body (Golgi ribbon) and in the proximal branchpoints of the dendritic tree (Golgi outposts), but are lacking from axons. By contrast, in addition to being present in the cell body, IC elements and REs are found throughout the neuronal periphery. The blow-up highlights synapses with local secretory ERES-IC-RE units. Whether axons contain similar structures is presently unclear. **(B)** Schematic diagram of a small portion of a long multinucleated *skeletal muscle cell.* In a terminally differentiated myofiber small Golgi outposts are found in the nuclear periphery and – together with ERES – at specific sites within the myofibrillar system. These sites, which function in the nucleation of longitudinal and vertical bundles of non-centrosomal (Golgi-nucleated) MTs most likely contain also IC elements and REs. **(C)** In a polarized *epithelial cell* the tight junctions divide the PM into apical and basolateral domains. The apical PM further consists of ciliary and non-ciliary subdomains. In epithelial cells, as in neurons and muscle cells, the centrosome loses its major role as MT-organizing center and (in this case) forms a basal body at the base of the primary cilium. This function is taken over by sub-apical nucleation sites which, however, remain enigmatic. These sites generate a vertical array of MTs typical for the polarized epithelial cell. The apical region may also contain a lateral array of MTs of mixed polarity (not shown). Moreover, a sub-population of non-centrosomal MTs are nucleated by the Golgi apparatus and grow apically. Rab11-containing apical recycling endosomes (AREs) pile-up at the minus ends of the vertical MTs. Similarly as in the pericentrosomal region of a fibroblastic cell (see Figure 4), a pool of IC elements are proposed join the REs at this location. This conclusion is supported by the existence of a circular membrane compartment at the base of the primary cilium, which is known to contain Rab11 and the IC/*cis*-Golgi protein GM130.

Cell differentiation requires major changes in the organization of endomembranes and cytoskeletal filaments, which frequently involve repositioning of the centrosome and the Golgi apparatus, as well as fragmentation of the continuous Golgi ribbon (Yadav and Linstedt, 2011; Sanchez and Feldman, 2017; Wei and Seemann, 2017). Another common denominator of neurons, epithelial and muscle cells is that in the course of their differentiation the centrosome loses its role as the major site of MT nucleation. While in some situations this function is taken over by the Golgi apparatus, in other cases the non-centrosomal sites of MT nucleation remain enigmatic (Nishita et al., 2017). In the following we address some of the subcellular rearrangements that accompany the differentiation of these three cell types, with special focus on the IC and endosomal networks and their proposed role in defining Golgi positioning (Figure 5).

### Neurons

The repositioning of the centrosome and the Golgi ribbon within the cell body plays a key role in the early stages of neuronal differentiation. Initially, this process is important for axon specification by ensuring polarized trafficking to the developing axon (de Anda et al., 2005), but it is also required for the formation of dendrites (Horton and Ehlers, 2004). In many neuronal cells the somatic Golgi ribbon faces the primary dendrite and even enters its proximal portion. However, the formation of Golgi outposts (GOPs) at the first branchpoints of the primary dendrite may not be due to dispersal of Golgi ministacks from the cell body, but rather to the movement of the IC elements and REs toward the cell periphery, in this case the growth cones of developing axons and dendrites (Sannerud et al., 2006; Eva et al., 2010; Matsuzaki et al., 2011; Figure 5A). This idea is supported by live imaging studies showing that thick tubules containing *cis*- or *trans*-Golgi markers move from the cell body to the dendrite, evidently having the capacity to form the GOPs (Quassollo et al., 2015). Of note, the latter have also been implicated in the formation of the dendritic MT network consisting of filaments of variable polarity (Ori-McKenney et al., 2012; Figure 5A).

In contrast to GOPs, the IC elements and REs are present throughout the dendritic tree (Hanus et al., 2014; Bourke et al., 2018; Figure 5A). At the level of the synapses these elements associate with ERES to establish local secretory units, also referred to as “secretory satellites” (Gardiol et al., 1999; Pierce et al., 2001; Hanus and Ehlers, 2016; Bowen et al., 2017; Figure 5A, inset). Strikingly, it turns out that hundreds of locally synthesized transmembrane glycoproteins – including neurotransmitter receptors, ion channels and neuronal adhesion proteins – can reach the synaptic PM with their glycans in the high-mannose form. These proteins employ a BFA-resistant Golgi bypass route across the ERES-IC-RE units (Hanus et al., 2016; Bowen et al., 2017), which has also been suggested to include Golgi-like components (Mikhaylova et al., 2016). Nonetheless, these findings open the possibility that Golgi bypass is not limited to the transport of selected proteins under special circumstances, but represents a basic mechanism for cell surface delivery of proteins and lipids (Prydz et al., 2013). In supporting dendritic compartmentalization and synaptic function, the IC elements and REs could also act as sites of MT nucleation (Figure 5A), since γ-tubulin is found throughout the dendritic tree, while GOPs are restricted to its proximal parts (Nguyen et al., 2014).

Interestingly, in *Drosophila* neurons mutations in genes encoding key transport machinery proteins – Sec23 (COPII), Sar1 and Rab1 – cause defects in dendritic rather than axonal morphology, showing that the growing dendrites preferentially depend on a functional early secretory pathway (Ye et al., 2007). Indeed, axons do not contain Golgi outposts (González et al., 2018), pointing to the possibility that the early secretory compartments (ERES and IC) in axons and dendrites differ in their overall organization or activity. Similarly, overexpression of GRASP65 exerts a preferential effect on the outgrowth of dendrites (Horton et al., 2005). Since GRASP65 most likely localizes to the IC and could even mediate the connection between the IC and endosomal networks (see above), its overexpression not only causes fragmentation of the Golgi ribbon in the cell body, but may lead to dysfunction of the IC elements at the neuronal periphery.

### Skeleletal Muscle Cells

During myogenesis, as mononuclear myoblasts differentiate into multinuclear myofibers, the centrosome undergoes dramatic reorganization as pericentriolar material – including the centrosomal proteins γ-tubulin and pericentrin – first redistributes to the periphery of the nuclei and then to a multitude of sites throughout the cell body (Tassin et al., 1985a; Zaal et al., 2011; Oddoux et al., 2013). The ensuing change in the pattern of MT nucleation is accompanied by fragmentation of the Golgi ribbon and a major reorganization of the Golgi stacks. While myoblasts contain a typical juxtanuclear Golgi next to the centrosome, during myogenesis Golgi elements first circle the nuclei and then are found as dispersed small cisternal stacks throughout the cell body (Ralston, 1993; Tassin et al., 1985b). However, like all membrane compartments in the skeletal muscle cells, the Golgi elements are also precisely positioned within the myofibrillar network, residing at the intersections of the longitudinal and vertical MT bundles that run across the cells (Figure 5B). Indeed, the Golgi elements have been implicated in the nucleation of the filaments that form of the stationery MT lattice typical for myofibers (Oddoux et al., 2013).

Currently, two alternatives have been considered regarding the nature of the ERES-Golgi units of myofibers. Either the Golgi ministacks emerging at these sites are formed *de novo*, due to recycling of Golgi components via the ER, or correspond to pre-existing elements derived from the fragmented Golgi ribbon that redistribute throughout the muscle cells (Lu et al., 2001; Zaal et al., 2011; Giacomello et al., 2019). As a compromise, we propose that repositioning of the permanent IC and endosomal networks provides the driving force for the rearrangement of the endomembrane system in muscle cells, including formation of the small Golgi stacks. First, there is evidence that both IC elements and endosomes are present at the same MT crossroads sites, where the Golgi elements reside (Rahkila et al., 1997; Kaisto et al., 1999; Figure 5B). Second, the IC/*cis*-Golgi proteins p115 and GM130 – both Rab1 effectors – have been shown to act as master regulators in the organization of early secretory compartments during myogenesis (Giacomello et al., 2019). Third, the nucleation of MTs in mature myofibers is not affected by BFA (Oddoux et al., 2013), raising the possibility that it is accomplished by the drug-resistant IC elements and REs.

### Epithelial Cells

Epithelial Madin-Darby canine kidney (MDCK) cells grown on filters initially show subcellular organization similar to that of fibroblasts, where the Golgi apparatus and the centrosome localize to one side of the nucleus, and the centrosome-nucleated MTs make up a radial array (Figure 4). Early 3-D studies employing confocal microscopy and EM revealed that as a tight and polarized epithelial monolayer is established, both organelles relocate to underneath the apical membrane (Bacallao et al., 1989; Buendia et al., 1990). At the same time, MTs reorganize into non-centrosomal, vertical arrays with their minus ends anchored in the apical region and plus ends pointing toward the basal part of the cell (Bacallao et al., 1989; Toya et al., 2016; Figure 5C). In addition, a subset of vertically oriented MTs nucleate at the Golgi membranes (Perez Bay et al., 2013; Figure 5C). Generally, the MT organization in different epithelial tissues varies considerably, involving a number of filament-associated proteins, such as CAMSAP3 (Toya and Takeichi, 2016).

Regarding endomembranes, while the outcome of epithelial differentiation on the endosomal system has been well characterized, its effects on early secretory compartments remain enigmatic. For example, it is unclear how the Golgi ribbon of polarized MDCK cells develops resistance to BFA. In the polarized state, separate pools of early endosomes operate in endocytic uptake at the apical and basolateral plasma membrane domains and the two routes meet in a common pool of late endosomes, localized – like lysosomes – on the apical side of the nucleus, with subsequent exchange of internalized cargo taking place within endosomes all around the nucleus (Bomsel et al., 1989). The apical endocytic machinery displays a relatively speaking much higher capacity of recycling and transcytosis of both fluid and membrane than its basolateral counterpart (Bomsel et al., 1989; Prydz et al., 1992). More recently, apical recycling endosomes (ARE) and common recycling endosomes (CRE) have been added to the picture, both positioned in the apical region on top of the nucleus (Leung et al., 2000). The latter most likely represents the compartment where fluid phase cargo endocytosed from the apical or basolateral surfaces first meet underway to late endosomes and lysosomes (Bomsel et al., 1989; Parton et al., 1989; Wang et al., 2000).

Thus, during differentiation of epithelial cells the endocytic apparatus splits into two systems serving the apical and basolateral domains of the cell. Accordingly, while in non-polarized MDCK cells different cell surface receptors follow the same recycling route via the Rab11-positive peri-centrosomal ERC, cell polarization involves the development of two compartments specialized into apical and basolateral recycling. Apparently based on its association with the centrosome, one of these compartments – the Rab11-positive ARE – moves to the sub-apical region, while the other – the Rab8-positive CRE, sharing compositional and functional similarity with the *trans*-Golgi/TGN – remains in the vicinity of the Golgi ribbon (Perez Bay et al., 2016). The permanent connections between the biosynthetic and endocytic networks observed in other cell types raise the possibility that the specialization of the two endocytic recycling circuits of epithelial cells is accompanied by a parallel “duplication” of the IC mediating membrane recycling at the ER-Golgi boundary (Figure 5C).

In polarized epithelial cells a primary cilium protruding from the apical membrane is anchored at the basal body, a structure consisting of the mother and daughter centrioles of the centrosome that during cell polarization relocated to the apical membrane, losing most of its pericentriolar material and ability to nucleate MTs (Figure 5C). Notably, newly synthesized proteins that are delivered to the ciliary membrane in a Rab11-, Rab8- and exocyst-dependent manner frequently follow pathways that bypass the Golgi stacks (Tian et al., 2014; Bernabé-Rubio and Alonso, 2017; Gilder et al., 2018; Witzgall, 2018). This transport is likely to involve a circular membrane compartment surrounding the base of the cilium that contains Rab11 and the IC/*cis*-Golgi protein GM130 (Kim et al., 2010; He et al., 2012; Stoops et al., 2015). Looking down from the apical side, the Rab11-positive AREs are normally found throughout the sub-apical region. Notably, however, upon knockout of CAMSAP3 – a protein with a key role in minus-end stabilization and anchoring of the vertical MTs – they accumulate around the basal body, which appears to regain the ability to nucleate a radial array of MTs (Noordstra et al., 2016; Toya et al., 2016). Finally, the periciliary compartment also contains Cdc42, which besides guiding centrosome repositioning during cell migration (see above) and epithelial polarization, is also required for ciliogenesis and ciliary protein trafficking (Wang et al., 2009; Bernabé-Rubio and Alonso, 2017).

## Summary and Perspectives

The present discussion focuses on two interconnected membrane systems, referred to as the biosynthetic and endocytic networks, which play key roles in membrane recycling in eukaryotic cells. Due to their ability to move bidirectionally along MT tracks, these membrane structures can assume wide cellular distributions and provide an essential link between the cell periphery and center in metazoans. Besides operating as a template for the biogenesis of the Golgi stacks, these networks may constitute a basic membrane system that plays an important role in trafficking and signaling events during different phases of the cell cycle; for example during cell division, when many trafficking events mediated by the classical protein coats (clathrin, COPI, COPII) appear to be compromised. Previously, the membranes meeting at the non-compact zones of the mammalian Golgi ribbon have been compared with the secretory system of the yeast *S. cerevisiae* (Marie et al., 2008; Jackson, 2009; Saraste and Marie, 2018).

The proposed model of the Golgi ribbon (Figure 4) is in accordance with the autonomous nature of the Golgi apparatus, as well as the ability of the cisternal stacks to form *de novo* (Emr et al., 2009). The linker compartments could represent a conserved, permanent aspect of the organelle and establish the basis for its autonomy. Moreover, the present model opens for communication across the Golgi stacks, possibly explaining events that occur on both sides of the organelle. One example is provided by Golgi-nucleation of MTs, which involves the formation of filaments at the *cis*-side of the stacks, and their stabilization at the *trans*-side (Efimov et al., 2007; Rivero et al., 2009). An interesting possibility is that the “hot-spots” observed in the nucleation of MTs (Sanders et al., 2017), as well as actin- and MT-dependent Golgi exit (Miserey-Lenkei et al., 2017), correspond to the non-compact zones of the Golgi ribbon. Another interesting case deals with autophagy, a complex process where both IC elements and REs have been implicated (Longatti and Tooze, 2012; Ge et al., 2013; Mochizuki et al., 2013; Puri et al., 2013). Again, it is possible that the linker compartments provide the “openings” that allow, for example, the transfer of key transmembrane protein Atg9 from the *trans*-Golgi/RE system via the IC to the site of autophagosome formation (Lamb et al., 2015; Imai et al., 2016; Mattera et al., 2017; Davies et al., 2018). Interestingly, the induction of autophagy results in the unlinking of the Golgi ribbon (Takahashi et al., 2011; Gosavi et al., 2018).

Regarding the various models on intra-Golgi trafficking, the present view on the organization of the Golgi ribbon (Figure 4) appears to be most compatible with the dynamic cisternal progression or maturation models, rather than the ones assuming vesicular transport between stable Golgi compartments (Glick and Luini, 2011). However, by providing a pathway for the passage of large-sized cargo molecules across the Golgi ribbon the present model of linker compartments would also be in line with the rim progression model of Rothman and coworkers, according to which the dilated rims and central portions of the stacks differ in their dynamics (Lavieu et al., 2013; 2014). Moreover, the proposed existence of permanent biosynthetic (pre-Golgi) and endocytic (post-Golgi) compartments at the two sides of the transient Golgi stacks could clarify a major discrepancy between the cisternal maturation model and the rapid-partitioning model based on the observation that the exit of various cargo from the Golgi typically follows exponential kinetics (Patterson et al., 2008). Thus, the permanent pre- and post-Golgi compartments could represent sites for the formation and fusion of the Golgi cisternae, respectively (Saraste and Kuismanen, 1992). Finally, according to the rapid-partitioning model the Golgi stacks are divided into processing and exit domains, a functional scenario that was previously incorporated in a two-domain structural model of the Golgi ribbon (Jackson, 2009), which has been elaborated further here.

Based on their permanent and dynamic nature, we propose here a dominant role for the biosynthetic and endocytic networks in Golgi positioning during cell division, migration and differentiation. In addition, this perspective is relevant for considering the events that take place in cells treated with MT-disruptive drugs, such as nocodazole, where the MT-based mobility of membranes is blocked and the Golgi ribbon is replaced by dispersed ministacks (Thyberg and Moskalewski, 1999). Two alternatives have been put forward to explain what happens during the drug treatment (when Golgi stacks are relocated to ERES) and wash-out (when a central Golgi ribbon is rapidly re-established). According to one the Golgi enzymes are temporarily redistributed to the ER (Cole et al., 1996a), whereas the other maintains that they remain within the Golgi proper (Pecot and Malhotra, 2006). Common to these models is that they both regard the Golgi stacks as mobile entities, which during drug wash-out can move along MTs to the cell center (Miller et al., 2009). The present considerations open a third possibility, namely that Golgi residents in both situations are redistributed to the biosynthetic (IC) and endosomal networks. How these networks receive them and also maintain their dynamics in the absence of MTs – possibly with the help of an actin-based system? – remain topics of future studies employing e.g., live imaging, super-resolution microscopy or correlative LM-EM.

Finally, the proposed model of the Golgi ribbon (Figure 4) prompts new thoughts regarding the development of endomembranes. Could it be that during evolution, to speed up trafficking and signaling between the periphery and center of the large-sized metazoan cells, their endomembrane system gradually established a connection with the newly arrived MT-system radiating from the centrosome. The centripetal movements of the primordial biosynthetic and endocytic compartments toward the centrosome, and their subsequently acquired capabilities to form cisternal stacks – representing an “annex” for efficient protein modification – could then explain the development of the ribbon-like organization of the Golgi in these cells. The dynamic connection between the Golgi ribbon and the centrosome also allowed the former to develop MT-nucleating activities over time. Consequently, the combined and variable roles of the centrosome and the Golgi in MT-nucleation could account for the diversity of Golgi structures seen in different metazoan cells. For example, lymphoid cells with a predominantly radial array of MTs form a circular Golgi around the centrosome, whereas fibroblasts displaying more active Golgi-based MT-nucleation build a more expanded and irregular perinuclear Golgi ribbon (Rios and Bornens, 2003). Highly differentiated cells again can employ and develop this toolbox to build amazing architectures to fulfill their functional needs (Figure 5).

Could the phylogeny of the Golgi ribbon be revealed when looking at the cells of early embryos? Strikingly, during early embryonic development in zebrafish, Golgi markers initially display a dispersed and punctate pattern. Around mid-gastrulation, the Golgi apparatus condenses in some cell types – like in the most superficial epithelial cells of the gastrula – evidently forming a ribbon, while other cell types maintain a dispersed pattern until later in development (Sepich and Solnica-Krezel, 2016). The morphological variation observed shows a potential correlation between more dispersed Golgi elements and a shorter cell cycle.

## Data Availability

All data analyzed for this study are included in the manuscript and the supplementary files.

## Author Contributions

Both authors confirm being the sole contributors of this work and have approved it for publication.

## Conflict of Interest Statement

The authors declare that the research was conducted in the absence of any commercial or financial relationships that could be construed as a potential conflict of interest.
